# Residency Preparation Courses: What Is the Durable Impact Beyond Medical School?

**DOI:** 10.1007/s40670-025-02453-1

**Published:** 2025-06-28

**Authors:** Rebecca C. Nause-Osthoff, Elizabeth K. Jones, Lauren A. Heidemann, Jessica L. Fealy, Samantha Kempner, Anita Malone, Zoe Stukenberg, Helen K. Morgan

**Affiliations:** 1 Department of Anesthesiology, Michigan Medicine, 4-911 Mott Hospital, 1540 East Hospital Drive, SPC 4245, Ann Arbor, 48109 MI USA; 2 Department of Family Medicine, Michigan Medicine, Ann Arbor, MI USA; 3 Department of Internal Medicine, Michigan Medicine, Ann Arbor, MI USA; 4 Department of Pediatrics, Michigan Medicine, Ann Arbor, MI USA; 5 Department of Obstetrics and Gynecology, Michigan Medicine, Ann Arbor, MI USA; 6 Department of Obstetrics and Gynecology, University of Utah, Salt Lake City, UT USA; 7 Learning Health Sciences, University of Michigan, Ann Arbor, MI USA

**Keywords:** Transition from medical school to residency, Transition to residency course, Residency preparation course, Medical student education, Curriculum

## Abstract

**Introduction:**

An increasing number of medical schools are implementing Residency Preparation Courses. Our objective was to examine learners’ perceptions of course impact 3 months into residency training—specifically, whether participation impacted patient care.

**Study design:**

An anonymous survey was sent electronically to individuals who completed a Residency Preparation Course at a single institution from 2020 to 2023. Participants rated their preparedness to start residency, their perceived preparedness compared to co-residents, and the perceived usefulness of various course components. Residents were asked if course content made a difference in patient care with the option to provide a free-text example.

**Results:**

Of 423 eligible individuals, 207 (48.9%) completed the survey. Overall, respondents rated their mean preparedness for residency as 3.8/5.0, and 4.0/5.0 when compared to their co-residents. Residents reported that inpatient paging, simulated mock codes, and procedural practice were the most useful curricular experiences. Overall, 94% of respondents somewhat or strongly agreed that something they learned in a Residency Preparation Course had already made a difference in patient care.

**Conclusions:**

This is one of the few studies that followed medical school graduates into residency, with 4 years of graduates who had a stable Residency Preparation Course curriculum, which was a graduation requirement. Notably, nearly all respondents reported that the course content improved their delivery of patient care. The historical chasm between medical school and residency must be bridged to improve this important educational transition for future physicians. This work highlights important learner perspectives on value added from Residency Preparation Courses.

**Supplementary Information:**

The online version contains supplementary material available at 10.1007/s40670-025-02453-1.

## Introduction

The transition from medical school to residency marks a critical juncture involving increased responsibility for patient care, abrupt changes in the type and setting of learning, intensified interprofessional collaboration, and ongoing professional development [1]. These demands come at a time of psychological vulnerability for trainees during their first few months of residency [2, 3]. Residency Preparation Courses (RPCs), also known as Transition to Residency Courses, are increasingly recognized as important curricular offerings to address the challenges associated with this transition [4]. As of the 2021–2022 academic year, over 150 medical schools currently offer some variation of RPC content [5].

Since implementing RPC curricula demands considerable time and effort, it is crucial to demonstrate the meaningful impact of this curricular addition. Multiple studies have described the benefits of RPCs [6–8]; however, evaluation data has often been limited to the Kirkpatrick Model evaluation levels of reaction (learner satisfaction and confidence) and learning (short-term skill acquisition) [9]. A few studies have followed learners into residency, with surveys of surgery residency program directors demonstrating perceived improvement in learners’ technical skills [10], clinical skills, and communication effectiveness [11]. One study of residents at a single institution demonstrated greater self-reported global preparedness as a resident [12] for learners who had participated in an RPC. Neither the specific educational components of RPCs that influence resident preparedness nor the potential benefits of RPC curricular content that represent higher levels of Kirkpatrick Model evaluation are well described. The lack of communication between undergraduate and graduate medical education likely contributes to the gap in understanding how best to address this important educational transition.

In order to determine learner perspectives at the transition to residency, our primary objective was to determine new residents’ perspectives on the value of RPC curricular components. We also sought to determine the impact of the RPC on patient care by asking open-ended reflective questions; these responses were analyzed, and common themes identified.

## Materials and Methods

### Setting and Participants

Participants were first-year residents who completed a specialty-specific RPC at our institution in February and March of 2020, 2021, 2022, or 2023. There are six specialty-specific RPCs available for medical students to complete during their final year of medical school, ranging from 4 to 8 weeks in duration: internal medicine, pediatrics, family medicine, emergency medicine, procedures, and obstetrics and gynecology. Completion of one RPC is a graduation requirement. Each RPC contains the curricular elements outlined in Table 2. These educational interventions are tailored to the specialty of the RPC. During the RPC, students can opt in to receive a follow-up survey by providing their permanent email address.

As background, our institution is a US allopathic medical school affiliated with a large academic medical center. The medical school curriculum is unique in that the pre-clinical curriculum is condensed into the first year (the Scientific Trunk). Medical students complete clinical clerkships during the second year (the Clinical Trunk) and clinical electives during the third and fourth years (the Clinical Branches).

### Intervention

We sent an anonymous electronic survey to all residents in each year’s cohort who provided their permanent email addresses in October of 2020, 2021, 2022, and 2023—approximately 3 months after they began residency. No incentive was given for survey participation. The survey was developed by the study authors based on a review of the literature and by using the Kirkpatrick model, which is based on several learning theories including constructivism (reactions), cognitivism (knowing), behaviorism (behavior change), and social learning theory. The survey creators were content experts who consulted previous surveys pertaining to the transition to residency, which contributed to content validity. We then piloted the survey with medical students, residents, and faculty, which contributed to response process validity. The full 2023 survey is available in Online Resource 1.

### Outcomes Measured

We inquired about demographic information including RPC completed and current specialty. We asked about preparedness to start residency on a 5-point Likert scale (1 = not at all prepared, 5 = very well prepared) and perceived preparedness compared to co-residents (1 = less well prepared, 5 = much better prepared). We asked residents to rate the usefulness of various RPC components (1 = not at all useful, 5 = extremely useful). We asked if the RPC made a difference in patient care (1 = strongly disagree, 5 = strongly agree); if so, residents were prompted to offer a free-text example. Respondents could go back to review previous survey questions while completing the survey. Inductive thematic analysis was performed by two independent assessors (R.C.N. and H.M.K.) to categorize responses and subsequently identify themes from the free-text entries. These themes were shared with the remaining authors after initial analysis. The authors have diverse educational backgrounds and practice different specialties. In analyzing the data, we thoughtfully considered how our experiences in clinical and academic medicine might influence our perspectives.

This study was deemed exempt by the University of Michigan Institutional Review Board. Data was stored on a secured University of Michigan shared drive. Residents signed consent to participate in this study during their RPC, as well as to receive follow-up email surveys after the course was completed.

## Results

From 2020 to 2023, 627 medical students participated in an RPC at our institution; of these, 423 (67.5%) provided a permanent email address, 207 (48.9%) of whom completed the survey. Respondents spanned each one of the RPCs offered at our institution (Table 1).

**Table 1:** Respondents’ residency preparation course completed and specialty pursued, 2020–2023, *N* = 193

RPC course completed	*n* (%) of total respondents	Specialty pursued (*n*)
Emergency Medicine	14 (7.3)	Emergency Medicine (14)
Family Medicine	14 (7.3)	Family Medicine (14)
Internal Medicine	71 (36.8)	Internal Medicine (49) Ophthalmology (10) Neurology (7) Psychiatry (5) Radiation Oncology (3) Dermatology (2) Physical Medicine and Rehabilitation (2) Radiology (2) Medicine-Pediatrics (1) Anesthesiology (1) Other (1)
Obstetrics and Gynecology	22 (11.4)	Obstetrics and Gynecology (22)
Pediatrics	14 (7.3)	Pediatrics (14)
Procedures	39 (20.2)	Anesthesiology (15) Urology (7) General Surgery (8) Otolaryngology (3) Plastic Surgery (2) Orthopedic Surgery (2) Ophthalmology (1)
Both Pediatric + Internal Medicine	4 (2.1)	Medicine—Pediatrics (4)
Not answered	15 (7.8)	Internal Medicine (3) Pediatrics (3) Emergency Medicine (2) Anesthesiology (2) Neurology (1) Family Medicine (1) Orthopedic Surgery (1) General Surgery (1)

*RPC* Residency Preparation Course

Overall, new residents rated their mean (SD) preparedness for residency as 3.8 (0.9). When asked to compare themselves to their co-residents, they assessed their mean self-rated preparedness as 4.0 (0.6). Residents reported that the course prepared them well, with a mean of 4.4 (1.0). When asked about specific curricular components of the RPC, residents reported that inpatient paging, simulated mock codes, and procedural practice were the most useful educational experiences. Table 2 shows a description and mean (SD) for each curricular component, and Table 3 includes the mean (SD) ratings for the curricular components for each RPC.

**Table 2:** Residency preparation course curricular components and perceived value by first-year residents, 2020–2023, *N* = 193

RPC curricular component	Description	Overall value^a^ Mean (SD)
Simulated Mock Codes or Clinical Emergency	Students are divided into groups of 5–6 and each assigned to lead a simulated code scenario on a high-fidelity mannequin. Students receive feedback on clinical decision-making, as well as leadership and communication skills, by their peers and a faculty instructor	4.4 (1.3)
Inpatient Paging	Students receive 3–6 specialty-specific pages from Standardized Registered Nurses about an urgent or emergent event on a simulated hospitalized patient for whom the student is provided a sign-out. The student calls the nurse and communicates diagnostic work-up and treatment decisions. The nurse grades the student on a standardized rubric and delivers formative verbal and written feedback about medical decision-making and communication	4.2 (1.7)
Procedural Practice	Students practice procedures that are pertinent to their intended specialty on both low-fidelity and high-fidelity mannequins in the simulation center, as well as in the anatomy lab (Procedures RPC). Examples include open and laparoscopic surgical skills, point of care ultrasound, insertion of central lines, placement of nasogastric tubes, vaginal laceration repair, and obstetric ultrasound. Assessment and feedback modalities vary by session	3.9 (1.5)
Outpatient Paging	Students receive 2–4 specialty-specific pages from simulated clinic patients who request an after-hours phone call about a common outpatient condition. Students are graded on a standardized rubric and given formative verbal and written feedback about medical decision-making and communication	3.5 (1.8)
Teaching a Topic to my Peers	Students receive didactic training about strategies to deliver an effective ‘chalk talk’ and then practice delivering 1–2 talks during the course to a group of their peers. After the session, the students receive formative feedback about their teaching effectiveness from their peers and a faculty instructor	3.2 (1.4)
Individualized Learning Plan	Students are asked to reflect on their performance during medical school (including review of prior evaluations) and develop an individualized learning plan moving forward that addresses specific areas for improvement and/or targeted goals for the residency transition. They are provided with example goals and are encouraged to develop one goal that is related to diversity, equity, and inclusion	2.6 (1.6)

^a^Overall value: Based on the question “Please rate how useful the following activities within the RPC were for preparing you for intern year” (1 = not at all prepared, 5 = extremely prepared)

*RPC* Residency Preparation Course

**Table 3:** Perceived value of residency preparation course curricular components by first-year residents, 2020–2023, *N* = 193

Component	Perceived usefulness^a^ of each RPC curricular component					
	**Emergency Medicine**	**Family Medicine**	**Internal Medicine**	**Obstetrics and Gynecology**	**Pediatrics**	**Procedures**^**b**^
Simulated mock code	4.9 (0.3)	3.5 (1.7)	4.6 (0.5)	4.5 (1.3)	4.5 (0.7)	3.6 (1.0)
Inpatient paging	3.1 (1.6)	3.1 (1.7)	4.6 (0.6)	4.1 (0.5)	4.3 (1.3)	4 (1.5)
Procedural practice	4.9 (0.2)	3.6 (1.1)	3.8 (2.2)	4.2 (1.7)	3.6 (1.0)	3.8 (0.9)
Outpatient paging	1.5 (0.8)	3.1 (1.7)	3.7 (1.6)	4 (0.8)	4 (2.4)	3.2 (1.6)
Teaching a topic to my peers	2.3 (0.3)	3.1 (1.3)	3.5 (1.4)	3.5 (1.0)	3.8 (0.9)	2.5 (1.5)
Individualized learning plan	2.9 (1.7)	2.7 (1.5)	2.4 (1.7)	3.0 (1.1)	2.6 (0.8)	2.2 (2.1)

Data presented as mean (SD)

^a^Usefulness of different RPC curricular elements were captured on a 5-point Likert scale (1 = not at all useful, 5 = extremely useful) and responses are collated by which course the respondent took

^b^The Procedures RPC is typically taken by students applying to procedural fields such as surgical specialties, anesthesiology, and interventional radiology

*RPC*, Residency Preparation Course

**Fig. 1:**
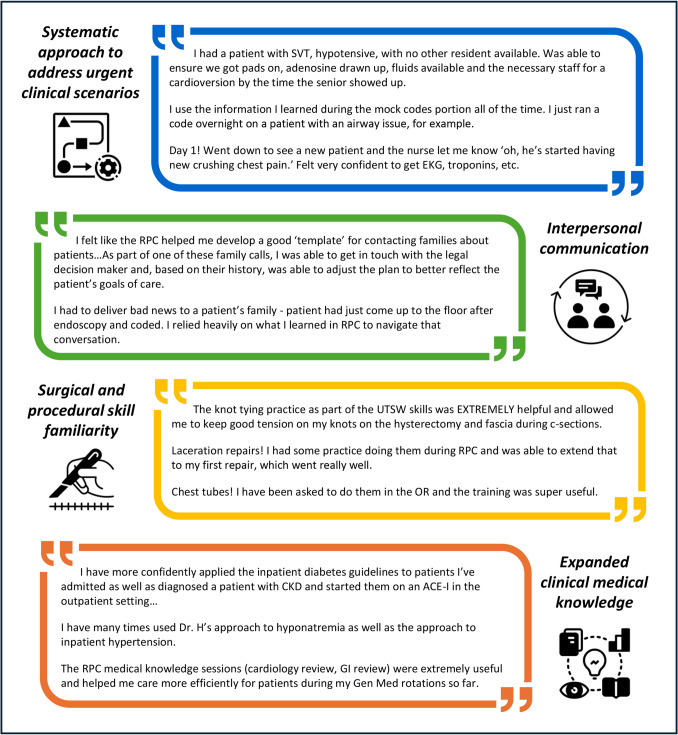
Qualitative analysis of narrative comments about impact of Residency Preparation Course on patient care during the first year of residency, 2020–2023, *N* = 123. Themes were identified in free-text entries by first-year residents based on the question, “What is an example of a time when something you learned from the RPC made a difference in patient care during your intern year?” Abbreviations: SVT, supraventricular tachycardia; EKG, electrocardiogram; RPC, Residency preparation course; UTSW, University of Texas Southwestern; OR, operating room; CKD, chronic kidney disease; ACE-I, angiotensin-converting enzyme I inhibitor; GI, gastrointestinal; Gen Med, general medicine

New residents were also asked to reflect on how the RPC has impacted patient care. Overall, 94% of respondents somewhat or strongly agreed that something they learned in the RPC had already made a difference in patient care. Qualitative analysis of narrative comments identified four common themes: systematic approach to address urgent clinical scenarios, interpersonal communication, surgical and procedural skill familiarity, and expanded clinical medical knowledge (Fig. 1).

## Discussion

This work demonstrates new residents’ perceived value of RPCs in the first few months of residency. Notably, nearly all respondents reported that the course content improved their delivery of patient care. This is one of the few studies that have followed medical school graduates into residency, with 4 years of graduates who had a stable RPC curriculum that was also a graduation requirement. By obtaining learner input during their transition to residency, we were able to demonstrate the durable value of RPCs through the Kirkpatrick Model evaluation level of behavior.

Given the acute stressors of increased patient care responsibilities during the transition to residency, it is not surprising that new residents valued content pertaining to urgent inpatient issues. Our findings are consistent with a recent survey of obstetrics and gynecology residents that demonstrated that residents felt they needed more preparation for management of inpatient issues [13]. In addition, a recent consensus recommendation on content for the transition to residency highlighted the assessment and management of common inpatient concerns [14]. Qualitative comments from our study’s respondents reflect how new residents appreciated learning schemas and systematic approaches to urgent scenarios. These findings highlight opportunities for the integration of inpatient curricular content that can be covered either in RPCs or new resident orientations.

Given the increased emphasis on building individual development plans (IDPs) at the transition to residency [15], it was notable and surprising how residents rated the IDP content much lower than other curricular items. Given that program directors cite self-reflection as one of the key areas of development for new interns [16], these findings demonstrate a disconnect in stakeholder opinion on this important issue. Because learners are likely focusing their attention on operational logistics of a new move [17], it will be imperative that we help them understand the relevance and usefulness of IDPs at this pivotal educational transition. An increased emphasis on an educational handover at the transition to residency may help learners understand the IDP in the context of the educational continuum between medical school and residency [18], as well as the importance of developing master adaptive learning skills [19] at the onset of their residency training.

For significant improvement to occur at the medical school to residency transition, meaningful input needs to be obtained from all stakeholders. This study adds value by following learners into residency; future work needs to continue to obtain feedback from stakeholders, including program directors, on curricular gaps and areas for development [20]. Limitations of this work are our reliance on learners’ subjective perspectives on what they needed for the transition to residency that were not corroborated by objective data on clinical performance and a potential response bias that may be present, as we obtained feedback only from individuals who provided us with their permanent email addresses. The historical chasm between learning and curricular content in medical school and residency needs to be bridged to improve this important educational transition for future physicians. This work highlights important learner perspectives on value added from RPC curricular content.

## Supplementary Information

Below is the link to the electronic supplementary material.

ESM 1 (PDF 274 KB)

## Data Availability

The data underlying this article will be shared on reasonable request to the corresponding author.
