# Supplementary material for: Residency Preparation Courses: What Is the Durable Impact Beyond Medical School?
Source: Med Sci Educ. 2025 Jun 28;35(5):2421–7. doi: 10.1007/s40670-025-02453-1 (PMC12812125; doi:10.1007/s40670-025-02453-1)
Supplement: Supplementary file 1 — (PDF 274 KB) [file 40670_2025_2453_MOESM1_ESM.pdf]

Online Resource 1. Residency Preparatory Course Intern Survey for 2023 Cohort, Completed by Respondents in Qualtrics

Medical Science Educator

Residency Preparation Courses: What is the Durable Impact Beyond Medical School?

Rebecca C. Nause-Osthoff, MD[1], Elizabeth K. Jones, MD[2], Lauren A. Heidemann, MD, MHPE[3], Jessica L. Fealy, MD[4], Samantha Kempner, MD[5], Anita Malone, MD[5], Zoe Stukenberg, MD[6], Helen K. Morgan, MD[5,7]

[1]Department of Anesthesiology, Michigan Medicine, Ann Arbor, MI USA
[2]Department of Family Medicine, Michigan Medicine, Ann Arbor, MI USA
[3]Department of Internal Medicine, Michigan Medicine, Ann Arbor, MI USA
[4]Department of Pediatrics, Michigan Medicine, Ann Arbor, MI USA
[5]Department of Obstetrics and Gynecology, Michigan Medicine, Ann Arbor, MI USA
[6]Department of Obstetrics and Gynecology, University of Utah, Salt Lake City, UT USA
[7]Learning Health Sciences, University of Michigan, Ann Arbor, MI USA

RPC Intern Survey 2023

Start of Block: Default Question Block

Q1 The purpose of this survey is to follow up to your participation in the University of Michigan Residency Preparation Course (RPC). Now that you have started your intern year, we would love to hear your perspective on how the RPC affected your experiences as an intern. We greatly appreciate you taking the time to respond to this short survey. Your responses will be used to improve the RPC curriculum and for scholarship. This survey will take approximately 5-10 minutes to complete. Your participation is anonymous and no identifying information will be collected. We hope that you are doing well, and forever Go Blue!

- ○ Click to write Choice 1 (4)
- ○ Click to write Choice 2 (5)
- ○ Click to write Choice 3 (6)

Page Break

Q2 What best describes your specialty?

- ○ Anesthesia (1)
- ○ Cardiothoracic Surgery (2)
- ○ Dermatology (3)
- ○ Emergency Medicine (4)
- ○ Family Medicine (5)
- ○ General Surgery (6)
- ○ Internal Medicine (7)
- ○ Medicine Pediatrics (8)
- ○ Neurology (9)
- ○ Neurosurgery (10)
- ○ Obstetrics & Gynecology (11)
- ○ Ophthalmology (12)
- ○ Orthopedic Surgery (13)
- ○ Otolaryngology (14)
- ○ Pathology (15)
- ○ Pediatrics (16)
- ○ Physical Medicine & Rehabilitation (17)
- ○ Plastic Surgery (18)
- ○ Psychiatry (19)
- ○ Radiation Oncology (20)
- ○ Radiology (21)

○ Surgery Integrated Program (22)

○ Urology (24)

○ Vascular Surgery (25)

○ Other (26) ____

Q3 Which Residency Preparation Course did you complete?

▢ Emergency Medicine (1)

▢ Family Medicine (2)

▢ Internal Medicine (3)

▢ Obstetrics & Gynecology (4)

▢ Pediatrics (5)

▢ Procedures (6)

Page Break

Q4 Reflecting back, please rate your preparedness to start intern year:

	Not at all prepared 1	2	3	4	Extremely prepared 5
()					

Q5 To the best of your estimation, please compare your preparedness for starting intern year with your co-interns:

	Not at all prepared 1	2	3	4	Extremely prepared 5
()					

Q6 Please rate how useful the following activities within the RPC were for preparing you for intern year:

	Not at all useful (1)	Slightly useful (2)	Moderately useful (3)	Very useful (4)	Extremely useful (5)	N/A (6)
Outpatient paging cases (1)	○	○	○	○	○	○
Inpatient paging cases (2)	○	○	○	○	○	○
Teaching a topic to my peers (i.e. chalk talk, power point, etc). (3)	○	○	○	○	○	○
Simulated mock codes and/or clinical emergency simulation (4)	○	○	○	○	○	○
Individualized learning goals/habit formation (5)	○	○	○	○	○	○
Procedural Practice/Ultrasound (6)	○	○	○	○	○	○
Patient-Initiated Harassment Session (8)	○	○	○	○	○	○
Overall course (7)	○	○	○	○	○	○

Display this question:

If Which Residency Preparation Course did you complete? = Internal Medicine

Q48 Please rate how useful the following activities within the IM RPC were for preparing you for intern year:

	Not at all useful (1)	Slightly useful (2)	Moderately useful (3)	Very useful (4)	Extremely useful (5)	N/A (6)
Delivering Serious News ("Vital Talk") to simulated actor (1)	○	○	○	○	○	○
Simulation day with Nurses (IV pump, fall precautions, pressure injury, restraints, NG tube, etc) (2)	○	○	○	○	○	○

Q10 What is something that was not included in the RPC course that you wished was included now that you are an intern?

____

____

____

____

____

Q32 During the RPC you received mock paging from nurses about inpatient emergencies (ex: afib w/ RVR, hyperkalemia, GI bleed, preterm labor, sepsis, mental status change, post-op pain, etc)

Please select which ACGME competencies the **RPC Paging Curriculum** made a difference in your transition to intern year. Select **ALL** that apply

- ☐ Patient care (1)
- ☐ Medical knowledge (2)
- ☐ Professionalism (3)
- ☐ Interpersonal and communication skills (4)

Page Break

Display this question:

If During the RPC you received mock paging from nurses about inpatient emergencies (ex: afib w/ RVR,... = Patient care

Or During the RPC you received mock paging from nurses about inpatient emergencies (ex: afib w/ RVR,... = Medical knowledge

Or During the RPC you received mock paging from nurses about inpatient emergencies (ex: afib w/ RVR,... = Professionalism

Or During the RPC you received mock paging from nurses about inpatient emergencies (ex: afib w/ RVR,... = Interpersonal and communication skills

Q36 If you can think of any *specific examples* of how the **RPC Paging Curriculum** helped you with any of these competencies we would love to hear it!

Display this question:

If During the RPC you received mock paging from nurses about inpatient emergencies (ex: afib w/ RVR,... = Patient care

Q37 What is an example of a time when something you learned from the **RPC Paging Curriculum** made a difference in **Patient Care** during your intern year?

____

____

____

____

____

Display this question:

If During the RPC you received mock paging from nurses about inpatient emergencies (ex: afib w/ RVR,... = Medical knowledge

Q38 What is an example of a time when something you learned from the **RPC Paging Curriculum** made a difference in **Medical Knowledge** during your intern year?

____

____

____

____

Display this question:

If During the RPC you received mock paging from nurses about inpatient emergencies (ex: afib w/ RVR,... = Professionalism

Q39 What is an example of a time when something you learned from the **RPC Paging Curriculum** made a difference in **Professionalism** during your intern year?

Display this question:

If During the RPC you received mock paging from nurses about inpatient emergencies (ex: afib w/ RVR,... = Interpersonal and communication skills

Q40 What is an example of a time when something you learned from the **RPC Paging Curriculum** made a difference in **Interpersonal and Communication Skills** during your intern year?

Page Break

Q34 Please rate your level of agreement with the following statement: I used something I learned during the RPC in internship that made a difference in patient care.

- Strongly disagree (8)
- Somewhat disagree (9)
- Neither agree nor disagree (10)
- Somewhat agree (11)
- Strongly agree (12)

Display this question:

If Please rate your level of agreement with the following statement: I used something I learned duri... = Somewhat agree

Or Please rate your level of agreement with the following statement: I used something I learned duri... = Strongly agree

Q35 What is an example of a time when something you learned from the **RPC** made a difference in patient care during your intern year?

____

____

____

____

____

Page Break

Q44 When you have experienced or witnessed inappropriate behaviors from patients as an intern, how often did you respond with skills introduced in the session on Patient-Initiated Harassment, Discrimination, Microaggressions?

	Not Applicable
	0 10 20 30 40 50 60 70 80 90 100
% ()	

Q45 Do you remember receiving a badge backer/PDF during the session on Patient-Initiated Harassment, Discrimination, Microaggressions?

○ Yes (1)

○ No (2)

Display this question:

If Do you remember receiving a badge backer/PDF during the session on Patient-Initiated Harassment,... = Yes

Q46 How often have you referenced the badge backer/PDF since starting residency?

____

Q47 Based on your experience as an intern thus far, what would you recommend be continued/added/removed from the session on Patient-Initiated Harassment, Discrimination, Microaggressions for future RPCs?"

____

____

____

____

____

Q12 Did you create an individualized learning goal or specific habit related to intern year?

- Yes (23)
- No (24)

Display this question:

If Did you create an individualized learning goal or specific habit related to intern year? = Yes

Q15 How much progress have you made toward your goal or habit?

- Have not started (1)
- A little progress (2)
- A lot of progress (3)
- Completed goal/habit is now part of regular routine (4)

End of Block: Default Question Block
